# Case Report: Anti-GABA_**A**_ Receptor Encephalitis in a Dog

**DOI:** 10.3389/fvets.2022.886711

**Published:** 2022-06-23

**Authors:** Enrice I. Huenerfauth, Christian G. Bien, Corinna Bien, Holger A. Volk, Nina Meyerhoff

**Affiliations:** ^1^Department of Small Animal Medicine and Surgery, University of Veterinary Medicine Foundation, Hannover, Germany; ^2^Laboratory Krone, Bad Salzuflen, Germany

**Keywords:** autoimmune encephalitis, GABA_A_ receptor encephalitis, seizures, epilepsy, dog

## Abstract

Autoantibodies against neurotransmitter receptors detected in cerebrospinal fluid (CSF) and serum are increasingly recognized in people with human autoimmune encephalitis causing severe neurological deficits, such as seizures and behavioral abnormalities. This case report describes the first encephalitis associated with antibodies against the γ-aminobutyric acid-A receptor (GABA_A_R) in a dog. A young male intact Cavalier King Charles Spaniel was presented with recent onset of initial multiple generalized tonic-clonic seizures progressing into a status epilepticus. Interictally, he showed alternating stupor and hyperexcitability, ataxia, pleurothotonus and circling behavior to the left side. Magnetic resonance imaging (MRI) of the brain showed breed-specific anatomical abnormalities. Standard CSF analysis was unremarkable. Despite treatment with multiple antiseizure medications (ASMs) seizures and behavior abnormalities sustained. Immunotherapy with dexamethasone was started on the fifth day after disease manifestation. This led to rapid improvement of clinical signs. An extensive antibody search in CSF and serum demonstrated a neuropil staining pattern on a tissue-based assay compatible with GABA_A_R antibodies. The diagnosis was confirmed by binding of serum and CSF antibodies to GABA_A_R transfected Human Embryonic Kidney cells. The serum titer was 1:320, the CSF titer 1:2. At the control visit 4.5 weeks after start of immunotherapy, the dog was clinically normal. The GABA_A_R antibody titer in serum had strongly decreased. The antibodies were no longer detectable in CSF. Based on clinical presentation and testing for GABA_A_R binding antibodies, this describes the first veterinary patient with an anti-GABA_A_R encephalitis with a good outcome following ASM and corticosteroid treatment.

## Introduction

Inflammatory autoimmune encephalopathies play a major role in canine neurology (1). They are described with the umbrella term meningoencephalitis of unknown origin (MUO), referring to a group of heterogenous sterile autoimmune encephalitides including the main histopathologically distinguishable subtypes: granulomatous meningoencephalitis (GME), necrotizing meningoencephalitis (NME) and necrotizing leucoencephalitis (NLE) (2, 3). Initiating trigger factors or underlying etiologies are largely unknown and a definite diagnosis is only possible with histopathological investigations of brain tissue (3).

In contrast, there is growing knowledge on autoimmune encephalitis in humans: In the last 40 years, a steadily increasing number of antibodies against neural intracellular or surface antigens in human patients with autoimmune encephalitis, often presenting with seizures and also psychiatric or behavior symptoms, have been described (1, 4, 5). Different subtypes of autoantibody associated encephalitis tend to differ in their initial clinical signs (6–8). As such autoantibodies bind to these antigens and reduce the number of accessible target receptors, a direct pathogenic influence is strongly suspected (9, 10). This increasing number of autoimmune encephalitis in Western countries is comparable to that of infectious ones (11, 12). In dogs as well, autoimmune inflammatory diseases have recently been described to outnumber infectious causes of CNS inflammation in the UK (13).

γ-aminobutyric acid-A receptors (GABA_A_R) are pentameric intrinsic or synaptic chloride ion channels and function as an inhibitory system of the postsynaptic potential in the brain (8, 10). Genetic mutations or aberrations due to antibodies binding to subunits (α1, β3, γ2) lead to a specific reduction of synaptic GABA_A_R, possibly inducing hyperexcitability and risk of seizures up to status epilepticus (6, 8, 14, 15). The disease appearance can depend on the affected subunit and the number of antibodies (6, 8, 16). Further symptoms described in humans can be personality and behavior changes, reduced consciousness, memory deficits or asymmetric signs like hemibody paresthesia (14, 16).

Case numbers of antibody associated encephalopathies are overall still low in human medicine and even more scarce in veterinary medicine (1, 17). Baulac et al. (18) already provided evidence of an association between GABA_A_R dysfunction and epilepsy in humans due to a mutation in the γ2-subunit gene in 2001. Nonetheless, GABA_A_R encephalitis is an only very recently reported disease in humans (19). In humans, a partial or complete recovery occurs in around 80% of cases with immunotherapy and early start of therapy improves prognosis (1, 8, 14). In dogs, so far only anti-*N*-methyl-D-aspartate receptor (NMDAR) 1 antibodies but no anti-GABA_A_ antibodies were detected in two dogs with MUO (17).

This case report describes the clinical signs, diagnostic work up and outcome of the first canine anti-GABA_A_R encephalitis in veterinary medicine.

## Case Description

An 1-year-old male intact Cavalier King Charles Spaniel (CKCS) was presented to the emergency service with a history of acute generalized tonic-clonic epileptic seizures progressing to status epilepticus. The owners reported acute onset of behavioral changes including unusual reactivity toward other dogs, restlessness, hyperesthesia when touched and excessive circling to the left, starting 2 days prior to presentation. The hyperexcitability was associated with anxiety and defensive aggression behavior when the dog was touched on different body parts like tail or pinnae. The dog was reported to be otherwise healthy except for a suspected food intolerance with recurrent episodes of diarrhea. He was fed with a hypoallergenic commercial diet. There was no previous travel history or a recent vaccination. The owner excluded the possibility of toxin ingestion as the dog was kept indoors and on the leash during walks.

On general examination immediately after the seizure events, the dog had an elevated body temperature of 39.7°C and a systolic cardiac murmur grade II of VI. The scrotal and inguinal skin was hyperemic.

The dog had severely reddened, moderately swollen conjunctives with unilateral subconjunctival hemorrhage and bilateral prolapse of the nictitating membranes. Ophthalmic examination revealed diffuse conjunctival chemosis in both eyes and a diffuse subconjunctival bleeding of the left eye.

The neurological examination was performed postictally. The dog showed a reduced level of mentation, a moderate head turn to the left, generalized ataxia and circling to the left. The dog reacted to touch with hyperexcitability and dysphoric vocalization, as well as compulsive circling to the left. Conscious proprioceptive placing was reduced on all four limbs. The menace response was absent and visual function was reduced. In the neuroanatomical localization a diffuse forebrain lesion potentially lateralized toward the left was suspected because of the asymmetrical clinical signs.

Hematology revealed a mild to moderate leukocytosis and a macrocytic thrombocytopenia. Serum biochemistry, glucose, clotting times, dynamic bile acid levels, urine analysis and blood pressure measurements were unremarkable.

Further examination was performed under general anesthesia. Premedication consisted of 0.15 mg/kg intravenous (IV) midazolam (Midazolam ratio 5 mg/ml, Ratiopharm GmbH, Ulm, Germany) and 0.3 mg/kg IV butorphanol (Butorgesic 10 mg/ml, CP-Pharma, Germany) as well as 0.6 mg/kg IV narcofol (Propofol 10 mg/ml, CP-Pharma, Germany). After endotracheal intubation, isoflurane E_T_ 0.9–1.3 Vol% (Isofluran CP, CP-Pharma, Germany) in oxygen was applied to maintain general anesthesia.

Magnetic resonance imaging (MRI, Achieva SmartParth to dStream for XR, a 3.0 T scanner, Philips Medical Systems, Best, The Netherlands) of the head depicted a mild Chiari-like malformation with flattening of frontal lobe with increased height of occipital lobe, suspected reduced volume of the caudal cranial fossa and caudal displacement of the cerebellum through the foramen magnum as well as a kinking of the craniocervical junction (20). No abnormal findings regarding inflammatory diseases or reversible postictal hyperintensities could be found in T2-weighted (w) Dixon or Fluid-attenuating inversion recovery (FLAIR), nor in T1w pre-and post-contrast, and susceptibility-weighted imaging sequences (17, 21).

The results of the cerebrospinal fluid (CSF) analysis obtained by cisternal puncture (nucleated cell count, total protein, cytology) were within normal reference ranges. The computed tomography (CT (IQon/Spectral CT, Philips GmbH, Hamburg, Germany) of the thorax revealed a markedly hypoattenuating area next to the tricuspid valve without contrast enhancement. An artifact, turbulence, injected gas or a thrombus was suspected. Repeated echocardiography (first under anesthesia and later when awake) did not confirm any cardiac abnormalities except for mitral regurgitation.

In the 2 days after general anesthesia, the dog remained stuporous with episodes of hyperexcitability. Seizure activity and transient obsessive circling to the right continued despite treatment with ASMs such as diazepam followed by phenobarbital. For seizure management, 2 mg/kg IV phenobarbital (Luminal, Sanacorp Langenhagen, Germany) was administered twice daily and during cluster seizures 2 mg/kg IV diazepam (Diazepam 10 mg/2 ml, B. Braun, Germany) and additional boli of 2 mg/kg IV phenobarbital. It was noticed that the epileptic seizures were triggered by stress during handling or touch.

To ensure nutritional supply, a parenteral gastrostomy tube was placed under general anesthesia with a premedication consisting of 4.8 μg/kg IV fentanyl (fentanyl 0.1 mg/2 ml, Sanacorp Langenhagen, Germany) and 0.5 mg/kg IV narcofol (Propofol 10 mg/ml, CP-Pharma, Germany) and isoflurane E_T_ 0.9–1.3 Vol % (Isofluran CP, CP-Pharma, Germany) in oxygen via an oxygen mask was applied to maintain general anesthesia.

Due to the lack of improvement, intravenous therapy with 0.5 mg/kg IV dexamethasone (Dexamethason, CP-Pharma, Germany) was started 5 days after clinical manifestation followed by 0.2 mg/kg IV. Gradual clinical improvement occurred over 3 days after start of dexamethasone administration. Then, the level of mentation and the behavior had improved significantly and were close to normal. His responsiveness to stimuli was still limited but he was aware of his surroundings and was interacting. At this timepoint, the dog showed a normal response to touch and handling rather than hyperexcitability or stupor as seen at presentation. The menace response deficits resolved. However, the head turn to the left and intermittent circling were still present. After 10 days of hospitalization, the dog was discharged with normal mentation, but still recurring intermittent circling to the left and bilateral proprioceptive deficits in the pelvic limbs.

Further examinations for possible pathogens such as tick borne encephalitis immunoglobulin G (IgG) antibodies enzyme-linked immunosorbent assay in CSF, Borna virus polymerase chain reaction (PCR) in ethylenediaminetetraacetic acid blood, fecal examination for parasites by flotation and emigration method according to Baermann, Angiostrongylus vasorum antigen and Anaplasma phagozytophilum PCR in serum were unremarkable.

**Figure 1:**
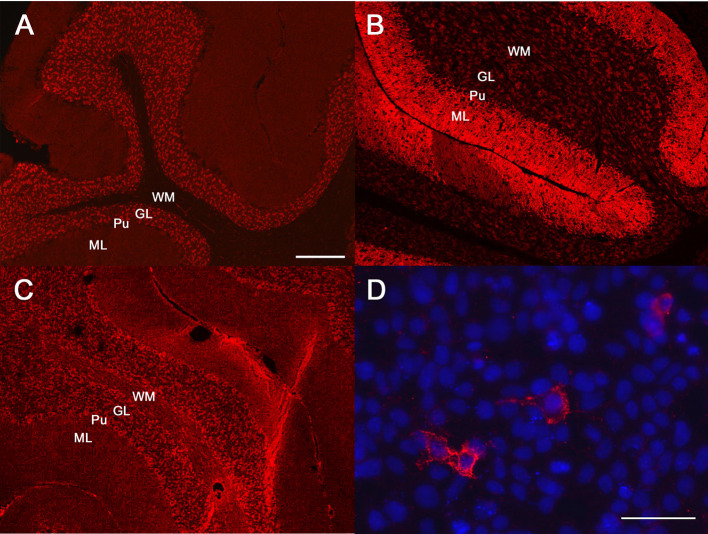
Antibody diagnostics. Immunofluorescence studies: diluted patient material is incubated with mouse brain sections or human embryonic kidney (HEK) cells transfected with the antigens of interest. Binding of patient antibodies to the respective matrix is visualized by a secondary anti-human/canine antibody coupled with an immunofluorescence dye resulting in a red signal. **(A–C)** Tissue-based assays, mouse cerebellum. **(D)** cell-based assay with HEK cells which were transfected with the γ-aminobutyric acid (GABA)-A receptor (GABA_A_R) subunits α1β3 (all assays from Euroimmun, Lübeck, Germany). Molecular layer (ML), purkinje cell layer (Pu), granular cell layer (GL), white matter (WM). **(A)** Human antibodies against the GABA_A_R. **(B)** Human antibodies against the GABA_B_R. **(C)** The canine antibodies against the GABA_A_R. Please note that the binding pattern is similar to **(A)** and not to **(B)**. **(D)** The canine serum binds to GABA_A_R transfected HEK cells. Nuclear counterstaining with Hoechst 33342 in blue. Bar in **(A)**, valid also for **(B,C)**: 100 μm. Bar in **(D)**: 25 μm.

Due to acute onset of epileptic seizures associated with marked behavioral changes resembling a psychosis in people, panels of neural antibodies were measured in serum and CSF by coauthors CGB, CB. An extensive antibody profile demonstrated a neuropil staining pattern on a tissue-based assay compatible with GABA_A_R antibodies (Figure 1); the diagnosis was confirmed by binding of serum and CSF to GABA_A_R (α1β3 subunits) transfected Human Embryonic Kidney cells [all assays from Euroimmun, Lübeck, Germany, used with in-house-protocols; for the canine material, secondary anti-dog Immunoglobulin G (IgG) was used]. The serum titer was 1:320, the CSF titer 1:2. Antibodies against NMDAR, leucine-rich glioma inactivated protein 1 (LGI1), glutamic acid decarboxylase like γ-aminobutyric acid-B receptor (GABA_B_R), IgLON family member 5, α-amino-3-hydroxy-5-methyl-4-isoxazolepropionic acid receptor, dipeptidyl-peptidase-like protein-6, contactin-associated protein-2, glycin receptor, metabotropic glutamate receptor 5 and metabotropic glutamate receptor 1 tested negative.

The dog showed continuous improvement with ASMs and 1 mg/kg oral corticosteroids (Prednitab vet. 5 mg, CP-Pharma, Germany) once daily. He remained seizure free, and behavior normalized. Four and a half weeks after discharge, the dog was neurologically normal at physical examination. Serum GABA_A_R antibodies were found at 1:20, and CSF was negative.

At a follow-up telephone conversation 8 months post diagnosis, owners reported the dog to be clinically normal without recurrence of epileptic seizures or behavioral abnormalities except for separation anxiety. The dog remained treated with 1.7 mg/kg phenobarbital twice daily. Corticosteroid therapy was tapered after 7 months to 0.7mg/kg daily, but then the dog developed a bacterial urinary tract infection and the local veterinarian tapered the corticosteroid dosage further down to 0,1mg/kg daily without a relapse of clinical signs.

## Discussion

This case report describes a 1-year-old CKCS presented with a history of acute progressive forebrain signs. He showed generalized tonic-clonic epileptic seizures, episodic hyperexcitability alternating with episode of stupor, and intermittent left circling behavior. Examination for neural autoantibodies in serum and CSF revealed GABA_A_R antibodies. The dog improved on immunotherapy with corticosteroids, after the initial ASM treatment failed.

In the last years, the number of cases being affected by neural auto-antibodies has increased in veterinary medicine (12). A very famous case was Knut the polar bear from the Berlin Zoo who was post mortem diagnosed with anti-NMDAR encephalitis (22). This led to further investigations. An age dependence of NMDAR antibody seroprevalence was found in dogs, cats, rats, baboons, and rhesus macaques (23). Furthermore, a direct link between autoimmune limbic encephalitis in cats with seizures and LGI1 autoantibodies has been suspected for years (24, 25). Stafford et al. (17) detected NMDAR antibodies in the CSF of two dogs with MUO. In addition, in dogs with necrotizing encephalitis, antibodies against astrocytic glial fibrillary acidic protein were recognized (26, 27). However, encephalitis associated with GABA_A_R has not been described in dogs until now.

In contrast to anti-GABA_B_R encephalitis, in which mainly middle-aged human patients with epileptic seizures as core symptoms are affected (28), GABA_A_R encephalitis is seen in a broad age range, even having been diagnosed in babies with a few months of age (14, 19). In children, nearly all patients suffer from generalized epileptic seizures, some also have a movement disorder. A neoplasm can usually not be found in affected children. Elderly patients often have a neoplastic condition (14). The most common associated tumor is a thymoma (8, 14). Other predisposing diseases, which have been associated with anti-GABA_A_R encephalitis, are a herpes virus infection, myasthenia gravis or other immunocompromising disorders (8, 10, 14, 29). No underlying viral infection (10), inflammatory condition or malignant disease was found in the dog, which underwent extensive tumor staging.

Clinical signs with acute onset seizures progressing to status epilepticus paired with erratic behavioral changes match the typical clinical signs in humans (10, 15). The post-ictal presentation of the CKCS was atypical and exceeded behavioral changes and aggression known from dogs after seizures (24, 30, 31). The clinical presentation and the lack of response to ASMs with rather unremarkable diagnostics raised our suspicion for an antibody associated encephalopathy.

The MRI in the canine case was performed within 48 h after onset of clinical signs and only showed abnormal brain anatomy which is typical for the breed. The finding of Chiari-like malformation in this dog is considered incidental. The CKCS did not have clinical signs typical for Chiari-like malformation, such as recurring vocalization, chronic recurrent spinal pain and reduced activity (20, 32). Nearly 92% of the CKCS population are affected by Chiari-like malformation (33).

In people with GABA_A_R encephalopathy, T2-w (FLAIR) sequences often reveal multifocal uni- or bilateral cortical and subcortical hyperintensities mainly of the temporal lobes without contrast enhancement (14, 19). Less frequently, a subcortical oedema can be seen (10). In 11.5% of the cases, the MRI is unremarkable (14). MRI abnormalities are more common in GABA_A_R encephalitis patients with a high serum antibody titer (6). On average, a diagnosis in humans is made 2 months after symptom onset or in a time span of 1 week to 5 years (14). An unremarkable MRI has been reported in 26.6% of patients with GABA_B_R encephalitis (34, 35). Interestingly, in anti-NMDA-R encephalitis, unremarkable MRIs are described in 45% of patients (36, 37). The MRI in our case was undertaken early after onset of clinical signs and functional impairment of receptors leading to interruption of neurotransmission and therefore seizure activity might have preceded visible edema or increased vascularization and leakage of the blood brain barrier. A follow-up MRI was not performed due to financial constraints. Standard CSF studies were unremarkable in our case. CSF analysis in human patients with GABA_A_R encephalopathy can present with pleocytosis and increased protein but also can be inconspicuous in up to 40% of the cases (6, 14).

A possible cause for an unremarkable MRI and CSF examination could be pretreatment with anti-inflammatory drugs such as corticosteroids or non-steroidal anti-inflammatory drugs. This was, however, not reported in the current case. Another explanation could be lack of sensitivity of the MRI or CSF to detect subtle changes. In cases of a mild leptomeningitis, ependymitits or encephalitis MRI can be normal (38, 39). In meningoencephalomyelitis of unknown origin up to 25% of dogs can have an unremarkable MRI and CSF analysis (40–42). In cases of autoantibody encephalitis, clinical manifestations could be due to antibody-mediated dysfunction of receptors (43). The MRI may not be sensitive enough to detect the initial inflammatory process. At a later stage an advanced demyelination and abnormal vascularization could be depicted with an MRI. To investigate this theory, MRI could be repeated later in the disease process, adding diffusion tensor imaging sequences that have been shown to detect abnormalities in human NMDAR encephalitis (44, 45) when there are no T2w FLAIR changes. Another theory is that changes are not seen on MRI as they are purely functional and do not cause changes in structure.

In man, a combination of clinical signs and examination of the autoantibodies in the CSF and serum is recommended for a definite diagnosis, as well as an extended screening for other neural antibodies (8, 14). The therapy consists of ASMs and an immunosuppressive dose of corticosteroids, which is tapered down gradually after 4 weeks, and then in weekly steps for 6–12 months if there is constant clinical improvement (1). In human medicine, additional immunosuppressive medication, intravenous immunoglobulin, plasma exchange and rituximab are used (1, 6, 14).

After the start of the therapy with corticosteroids, the CKCS improved continuously. At the control visit 1 month later, GABA_A_R auto-antibodies have decreased significantly. When the antibody production can be stopped, in man, the titer decreases by 50% (one titration level) every 4 weeks (46). In our case, the titer decreased by four levels. Such a rapid decrease could be explained by a shorter half-life of IgG in dogs compared to humans; in fact, the canine IgG half-life is unknown (47). A laboratory inaccuracy [inadvertent deviations by 1 titer level are occasionally hard to avoid (48)] may have contributed to the rapid titer decline.

Interestingly, it was reported by the breeder that a relative of the dog from a previous litter also suffered of epileptic seizures and acute onset circling. Because of a fast and progressive course, the puppy died within hours. No diagnosis was available in this dog, but a familial predisposition for autoimmune brain disease could be possible (49, 50). Nevertheless, heritable basis has also been described for idiopathic epilepsy in CKCS (51).

Even if many parallels can be drawn between human and veterinary medicine with regard to the clinical signs and the corresponding auto-antibodies, it must still be questioned in principle whether the respective antibodies are causative. The syndrome was similar to that of affected humans. The antibody titer decreased in parallel to the clinical improvement. Finally, tests for other neural auto-antibodies gave negative results. All these arguments speak in favor of a pathogenic relevance of the GABA_A_R antibodies.

## Conclusion

We describe the first case of a dog with an anti-GABA_A_R encephalitis. Based on the clinical signs and presentation (epileptic seizures lacking any response to ASMs, erratic behavioral changes) and the results of the initial diagnostics (lack of abnormal findings on conventional MRI and CSF examination), an autoimmune encephalitis was suspected, proven, and successfully treated. Clinicians should consider to test for autoantibodies and start immunotherapy in cases with a similar clinical presentation and lack of response to anti-seizure medication even if an inflammatory/infectious or neoplastic cause was clinically excluded on MRI and CSF.

## Data Availability Statement

The raw data supporting the conclusions of this article will be made available by the authors, without undue reservation.

## Ethics Statement

Ethical review and approval was not required for the animal study because the case report describes normal routine clinical workup. Written informed consent was obtained from the owners for the participation of their animals in this study.

## Author Contributions

EH saw the case with input from NM and HV. ChB and CoB performed the autoantibodies analysis. ChB provided the figure. EH wrote the initial draft. All authors reviewed, revised, and approved the submitted version.

## Funding

This Open Access publication was funded by the Deutsche Forschungsgemeinschaft (DFG, German Research Foundation) within the programme LE 824/10-1 Open Access Publication Costs and University of Veterinary Medicine Hannover, Foundation.

## Conflict of Interest

The authors declare that the research was conducted in the absence of any commercial or financial relationships that could be construed as a potential conflict of interest. The reviewer HG declared a shared affiliation with the author HV to the handling editor at the time of the review.

## Publisher's Note

All claims expressed in this article are solely those of the authors and do not necessarily represent those of their affiliated organizations, or those of the publisher, the editors and the reviewers. Any product that may be evaluated in this article, or claim that may be made by its manufacturer, is not guaranteed or endorsed by the publisher.

## Abbreviations

ASM: antiseizure drugs medication
CKCS: Cavalier King Charles Spaniel
CSF: cerebrospinal fluid
FLAIR: fluid-attenuated inversion recovery
GABA_A_R: γ-aminobutyric acid-A receptors
GABA_B_R: γ-aminobutyric acid-B receptor
IgG: immunoglobulin G
IV: intravenously
LGI1: leucine-rich glioma-inactivated protein 1
MUO: meningoencephalitis of unknown origin
NMDAR: *N*-methyl-D-aspartate receptor
PCR: polymerase chain reaction
w: weighted.

## References

[1] Bien CG Holtkamp M. “Autoimmune epilepsy”: encephalitis with autoantibodies for epileptologists. Epilepsy Curr. (2017) 17:134–41. 10.5698/1535-7511.17.3.134 28684941 PMC5486416

[2] Paušová TK Tomek A Šrenk P Belašková S. Clinical presentation, diagnostic findings, and long-term survival time in 182 dogs with meningoencephalitis of unknown origin from central europe that were administered glucocorticosteroid monotherapy. Top Companion Anim Med. (2021) 44:100539. 10.1016/j.tcam.2021.100539 33964477

[3] Lowrie M Smith PM Garosi L. Meningoencephalitis of unknown origin: investigation of prognostic factors and outcome using a standard treatment protocol. Vet Rec. (2013) 172:527–527. 10.1136/vr.101431 23462382

[4] Irani SR Bien CG Lang B. Autoimmune epilepsies. Curr Opin Neurol. (2011) 24:146–53. 10.1097/WCO.0b013e3283446f05 21358545

[5] Zhang M Li W Zhou S Zhou Y Yang H Yu L. Clinical features, treatment, and outcomes among chinese children with anti-methyl-D-aspartate receptor (anti-NMDAR) encephalitis. Front Neurol. (2019) 10:596–596. 10.3389/fneur.2019.00596 31244759 PMC6562280

[6] Petit-Pedrol M Armangue T Peng X Bataller L Cellucci T Davis R. Encephalitis with refractory seizures, status epilepticus, and antibodies to the GABAA receptor: a case series, characterisation of the antigen, and analysis of the effects of antibodies. Lancet Neurol. (2014) 13:276–86. 10.1016/S1474-4422(13)70299-0 24462240 PMC4838043

[7] Kreye J Wright SK van Casteren A Stöffler L Machule ML Reincke SM. Encephalitis patient-derived monoclonal GABAA receptor antibodies cause epileptic seizures. J Exp Med. (2021) 218:e20210012. 10.1101/2021.01.28.428602 34546336 PMC8480667

[8] Quek AML O'Toole O. Encephalitis associated with autoantibodies binding to γ-aminobutyric acid-A, γ-aminobutyric acid-B and glycine receptors: immunopathogenic mechanisms and clinical characteristics. Neurol-Neuroimmunol. (2016) 3:86–92.

[9] Brändle SM Cerina M Weber S Held K Menke AF Alcalá C. Cross-reactivity of a pathogenic autoantibody to a tumor antigen in GABA(A) receptor encephalitis. Proc Natl Acad Sci USA. (2021) 118:e1916337118. 10.1073/pnas.1916337118 33619082 PMC7936355

[10] Lancaster E. Encephalitis, severe seizures, and multifocal brain lesions: Recognizing autoimmunity to the GABA(A) receptor. Neurol Neuroimmunol Neuroinflamm. (2019) 6:e554–e554. 10.1212/NXI.0000000000000554 31044145 PMC6467683

[11] Dubey D Pittock SJ Kelly CR McKeon A Lopez-Chiriboga AS Lennon VA. Autoimmune encephalitis epidemiology and a comparison to infectious encephalitis. Ann Neurol. (2018) 83:166–77. 10.1002/ana.25131 29293273 PMC6011827

[12] Prüss H. Autoantibodies in neurological disease. Nat Rev Immunol. (2021) 21:798–813. 10.1038/s41577-021-00543-w 33976421 PMC8111372

[13] Gonçalves R De Decker S Walmsley G Butterfield S Maddox TW. Inflammatory disease affecting the central nervous system in dogs: a retrospective study in England (2010–2019). Front Vet Sci. (2022) 8:819945. 10.3389/fvets.2021.819945 35155652 PMC8829331

[14] Spatola M Petit-Pedrol M Simabukuro MM Armangue T Castro FJ Barcelo Artigues MI. Investigations in GABA(A) receptor antibody-associated encephalitis. Neurology. (2017) 88:1012–20. 10.1212/WNL.0000000000003713 28202703 PMC5384834

[15] Pettingill P Kramer HB Coebergh JA Pettingill R Maxwell S Nibber A. Antibodies to GABAA receptor α1 and γ2 subunits: clinical and serologic characterization. Neurology. (2015) 84:1233–41. 10.1212/WNL.0000000000001326 25636713 PMC4366091

[16] Ohkawa T Satake S Yokoi N Miyazaki Y Ohshita T. Identification and characterization of GABA(A) receptor autoantibodies in autoimmune encephalitis. J Neurosci. (2014) 34:8151. 10.1523/JNEUROSCI.4415-13.2014 24920620 PMC6608235

[17] Stafford EG Kortum A Castel A Green L Lau J Early PJ. Presence of cerebrospinal fluid antibodies associated with autoimmune encephalitis of humans in dogs with neurologic disease. J Vet Intern Med. (2019) 33:2175–82. 10.1111/jvim.15616 31495976 PMC6766506

[18] Baulac S Huberfeld G Gourfinkel-An I Mitropoulou G Beranger A Prud'homme JF. First genetic evidence of GABA(A) receptor dysfunction in epilepsy: a mutation in the gamma2-subunit gene. Nat Genet. (2001) 28:46–8. 10.1038/ng0501-46 11326274

[19] O'Connor K Waters P Komorowski L Zekeridou A Guo C-Y Mgbachi VC. GABA(A) receptor autoimmunity: a multicenter experience. Neurol Neuroimmunol Neuroinflamm. (2019) 6:e552. 10.1212/NXI.0000000000000552 31119187 PMC6501640

[20] Knowler SP Galea GL Rusbridge C. Morphogenesis of Canine Chiari malformation and secondary syringomyelia: disorders of cerebrospinal fluid circulation. Front Vet Sci. (2018) 5:171. 10.3389/fvets.2018.00171 30101146 PMC6074093

[21] Mellema LM Koblik PD Kortz GD LeCouteur RA Chechowitz MA Dickinson PJ. Reversible magnetic resonance imaging abnormalities in dogs following seizures. Vet Radiol Ultrasound. (1999) 40:588–95. 10.1111/j.1740-8261.1999.tb00884.x 10608685

[22] Prüss H Leubner J Wenke NK Czirják GÁ Szentiks CA Greenwood AD. Anti-NMDA receptor encephalitis in the polar bear (Ursus maritimus) knut. Sci Rep. (2015) 5:12805. 10.1038/srep12805 26313569 PMC4551079

[23] Pan H Oliveira B Saher G Dere E Tapken D Mitjans M. Uncoupling the widespread occurrence of anti-NMDAR1 autoantibodies from neuropsychiatric disease in a novel autoimmune model. Mol Psychiatry. (2019) 24:1489–501. 10.1038/s41380-017-0011-3 29426955 PMC6756099

[24] Pakozdy A Halasz P Klang A Bauer J Leschnik M Tichy A. Suspected limbic encephalitis and seizure in cats associated with voltage-gated potassium channel (VGKC) complex antibody. J Vet Intern Med. (2013) 27:212–4. 10.1111/jvim.12026 23278981

[25] Hasegawa D Ohnishi Y Koyama E Matsunaga S Ohtani S Nakanishi A. Deleted in colorectal cancer (netrin-1 receptor) antibodies and limbic encephalitis in a cat with hippocampal necrosis. J Vet Intern Med. (2019) 33:1440–5. 10.1111/jvim.15492 30942925 PMC6524083

[26] Devinsky O Boesch JM Cerda-Gonzalez S Coffey B Davis K Friedman D. A cross-species approach to disorders affecting brain and behaviour. Nat Rev Neurol. (2018) 14:677–86. 10.1038/s41582-018-0074-z 30287906

[27] Matsuki N Fujiwara K Tamahara S Uchida K Matsunaga S Nakayama H. Prevalence of autoantibody in cerebrospinal fluids from dogs with various CNS diseases. J Vet Med Sci. (2004) 66:295–7. 10.1292/jvms.66.295 15107560

[28] Zhu F Shan W Lv R Li Z Wang Q. Clinical characteristics of anti-GABA-B receptor encephalitis. Front Neurol. (2020) 11:403. 10.3389/fneur.2020.00403 32508739 PMC7253677

[29] Graus F Titulaer MJ Balu R Benseler S Bien CG Cellucci T. A clinical approach to diagnosis of autoimmune encephalitis. Lancet Neurol. (2016) 15:391–404. 10.1016/S1474-4422(15)00401-9 26906964 PMC5066574

[30] Watson F Rusbridge C Packer RMA Casey RA Heath S Volk HA. review of treatment options for behavioural manifestations of clinical anxiety as a comorbidity in dogs with idiopathic epilepsy. Vet J. (2018) 238:1–9. 10.1016/j.tvjl.2018.06.001 30103909

[31] Packer RMA McGreevy PD Salvin HE Valenzuela MJ Chaplin CM Volk HA. Cognitive dysfunction in naturally occurring canine idiopathic epilepsy. PLoS ONE. (2018) 13:e0192182. 10.1371/journal.pone.0192182 29420639 PMC5805257

[32] Rusbridge C McFadyen AK Knower SP. Behavioral and clinical signs of Chiari-like malformation-associated pain and syringomyelia in Cavalier King Charles spaniels. J Vet Intern Med. (2019) 33:2138–50. 10.1111/jvim.15552 31290195 PMC6766577

[33] Olsen E Suiter EJ Pfau T McGonnell IM Matiasek K Giejda A. Cavalier King Charles Spaniels with Chiari-like malformation and Syringomyelia have increased variability of spatio-temporal gait characteristics. BMC Vet Res. (2017) 13:159. 10.1186/s12917-017-1077-5 28587601 PMC5461676

[34] Lancaster E Lai M Peng X Hughes E Constantinescu R Raizer J. Antibodies to the GABA(B) receptor in limbic encephalitis with seizures: case series and characterisation of the antigen. Lancet Neurol. (2010) 9:67–76. 10.1016/S1474-4422(09)70324-2 19962348 PMC2824142

[35] Kitazaki Y Ikawa M Yamaguchi T Enomoto S Kishitani T Shirafuji N. Autoimmune encephalitis associated with anti-gamma-aminobutyric acid B receptor antibodies mimicking syncope. Intern Med. (2020) 59:843–7. 10.2169/internalmedicine.3652-19 31813910 PMC7118379

[36] Dalmau J Gleichman AJ Hughes EG Rossi JE Peng X Lai M. Anti-NMDA-receptor encephalitis: case series and analysis of the effects of antibodies. Lancet Neurol. (2008) 7:1091–8. 10.1016/S1474-4422(08)70224-2 18851928 PMC2607118

[37] Titulaer MJ McCracken L Gabilondo I Armangué T Glaser C Iizuka T. Treatment and prognostic factors for long-term outcome in patients with anti-NMDA receptor encephalitis: an observational cohort study. Lancet Neurol. (2013) 12:157–65. 10.1016/S1474-4422(12)70310-1 23290630 PMC3563251

[38] Mathews VP Kuharik MA Edwards MK D'Amour PG Azzarelli B Dreesen RG. Dyke award. Gd-DTPA-enhanced MR imaging of experimental bacterial meningitis: evaluation and comparison with CT. Am J Roentgenol. (1989) 152:131–6. 10.2214/ajr.152.1.131 2783267

[39] Lobetti RG Pearson J. Magnetic Resonance Imaging in the diagnosis of focal granulomatous meningoencephalitis in two dogs. Vet Radiol Ultrasound. (1996) 37:424–7. 10.1111/j.1740-8261.1996.tb01254.x

[40] Bohn AA Wills TB West CL Tucker RL Bagley RS. Cerebrospinal fluid analysis and magnetic resonance imaging in the diagnosis of neurologic disease in dogs: a retrospective study. Vet Clin Pathol. (2006) 35:315–20. 10.1111/j.1939-165X.2006.tb00138.x 16967416

[41] Lamb CR Croson PJ Cappello R Cherubini GB. Magnetic resonance imaging findings in 25 dogs with inflammatory cerebrospinal fluid. Vet Radiol Ultrasound. (2005) 46:17–22. 10.1111/j.1740-8261.2005.00003.x 15693553

[42] Granger N Smith PM Jeffery ND. Clinical findings and treatment of non-infectious meningoencephalomyelitis in dogs: a systematic review of 457 published cases from 1962 to 2008. Vet J. (2010) 184:290–7. 10.1016/j.tvjl.2009.03.031 19410487

[43] Peer M Prüss H Ben-Dayan I Paul F Arzy S Finke C. Functional connectivity of large-scale brain networks in patients with anti-NMDA receptor encephalitis: an observational study. Lancet Psychiatry. (2017) 4:768–74. 10.1016/S2215-0366(17)30330-9 28882707

[44] Finke C. Diagnosing MRI-negative autoimmune diseases. Neurol Neuroimmunol Neuroinflamm. (2018) 5:e457. 10.1212/NXI.0000000000000457 29616234 PMC5880627

[45] Finke C Kopp UA Scheel M Pech LM Soemmer C Schlichting J. Functional and structural brain changes in anti-N-methyl-D-aspartate receptor encephalitis. Ann Neurol. (2013) 74:284–96. 10.1002/ana.23932 23686722

[46] Waldmann TA Strober W Blaese RM. Metabolism of immunoglobulins. In: Amos B editor. Progress in Immunology. Cambridge, MA: Academic Press. (1971), p. 891–903. 10.1016/B978-0-12-057550-3.50072-7

[47] Bergman D Bäckström C Hansson-Hamlin H Larsson A Holst BS. Pre-existing canine anti-IgG antibodies: implications for immunotherapy, immunogenicity testing and immunoassay analysis. Sci Rep. (2020) 10:12696. 10.1038/s41598-020-69618-3 32728049 PMC7391631

[48] Reiber H Lange P. Quantification of virus-specific antibodies in cerebrospinal fluid and serum: sensitive and specific detection of antibody synthesis in brain. Clin Chem. (1991) 37:1153–60. 10.1093/clinchem/37.7.1153 1855284

[49] Muñiz-Castrillo S Vogrig A Honnorat J. Associations between HLA and autoimmune neurological diseases with autoantibodies. Autoimmun Highlights. (2020) 11:2. 10.1186/s13317-019-0124-6 32127039 PMC7065322

[50] Greer KA Schatzberg SJ Porter BF Jones KA Famula TR Murphy KE. Heritability and transmission analysis of necrotizing meningoencephalitis in the Pug. Res Vet Sci. (2009) 86:438–42. 10.1016/j.rvsc.2008.10.002 19014875

[51] Rusbridge C Knowler SP. Inheritance of occipital bone hypoplasia (Chiari type I malformation) in Cavalier King Charles Spaniels. J Vet Intern Med. (2004) 18:673–8. 10.1111/j.1939-1676.2004.tb02605.x 15515584

